# Structure of the WipA protein reveals a novel tyrosine protein phosphatase effector from *Legionella pneumophila*

**DOI:** 10.1074/jbc.M117.781948

**Published:** 2017-04-07

**Authors:** Nikos Pinotsis, Gabriel Waksman

**Affiliations:** From the ‡ Department of Biological Sciences, Institute of Structural and Molecular Biology, Birkbeck, Malet Street, WC1E 7HX London, United Kingdom and; the § Institute of Structural and Molecular Biology, University College London, WC1E 6BT London, United Kingdom

**Keywords:** crystal structure, enzyme kinetics, Michaelis-Menten, phosphatase, tyrosine-protein phosphatase (tyrosine phosphatase), Legionella effector, coiled-coil, phosphoesterase fold, tyrosine phosphatase

## Abstract

Legionnaires' disease is a severe form of pneumonia caused by the bacterium *Legionella pneumophila. L. pneumophila* pathogenicity relies on secretion of more than 300 effector proteins by a type IVb secretion system. Among these *Legionella* effectors, WipA has been primarily studied because of its dependence on a chaperone complex, IcmSW, for translocation through the secretion system, but its role in pathogenicity has remained unknown. In this study, we present the crystal structure of a large fragment of WipA, WipA435. Surprisingly, this structure revealed a serine/threonine phosphatase fold that unexpectedly targets tyrosine-phosphorylated peptides. The structure also revealed a sequence insertion that folds into an α-helical hairpin, the tip of which adopts a canonical coiled-coil structure. The purified protein was a dimer whose dimer interface involves interactions between the coiled coil of one WipA molecule and the phosphatase domain of another. Given the ubiquity of protein-protein interaction mediated by interactions between coiled-coils, we hypothesize that WipA can thereby transition from a homodimeric state to a heterodimeric state in which the coiled-coil region of WipA is engaged in a protein-protein interaction with a tyrosine-phosphorylated host target. In conclusion, these findings help advance our understanding of the molecular mechanisms of an effector involved in *Legionella* virulence and may inform approaches to elucidate the function of other effectors.

## Introduction

Legionnaires' disease is a severe form of pneumonia first reported in 1976 during an American Legion convention in Philadelphia caused by the pathogen *Legionella pneumophila* (1). *Legionella* bacteria annually infect ∼15,000 people in the United States, and similar numbers are reported in Europe. Approximately 10% of these cases are lethal, particularly for older or weaker people (2). Nosocomial outbreaks of the disease are more severe, resulting in fatality rates up to 50% (3–5). Treatment may require antibiotics for several weeks up to years for chronic cases. Understanding the mechanisms by which *Legionella* strains cause infections is therefore important. Moreover, there are striking similarities between the pathogenicity mechanisms from *Legionella* and those from other bacterial pathogens such as *Coxiella burnetti*, *Mycobacterium tuberculosis*, and *Rickettsiella grylli* (6–8).

*Legionella* bacteria are ubiquitous and mainly parasitize freshwater protozoa (7). However, pathogenic strains can survive and replicate within alveolar macrophages after inhalation of aerosols (*e.g.* from air-conditioning systems, hot baths, showers) (9). Within the macrophage, the bacterium establishes a protected vacuole (*Legionella*-containing vacuole) that bypasses the usual phagosome maturation pathway and provides an adequate niche in which the bacterium can multiply and propagate (10). The most important virulence factor of *L. pneumophila* is a type IVb secretion system (T4bSS, also known as the Dot/Icm secretion system)^2^ (8). Proteins secreted by the T4bSS (also known as protein effectors or effectors) are directly involved in the construction of the *Legionella*-containing vacuole, and in general they interfere with a large variety of functions in the host organism (10, 11). Overall, ∼300 open reading frames have been identified as effectors secreted by the Dot/Icm system, which is ∼10% of the entire *L. pneumophila* genome (11). During the first step of protein translocation, specific chaperones (*e.g.* IcmSW, a chaperone consisting of two proteins, IcmS and IcmW) bind effectors and promote their secretion via the T4bSS. A number of effector proteins also appear to have C-terminal signal sequences that target them to the T4bSS, and many of these sequences are highly negatively charged, being composed of glutamate residues (E-blocks) (12). However, these block sequences are not universal, and the recruitment mode of effectors remains unclear (12, 13).

Here we describe the crystal structure and biochemical properties of WipA (or IcmW-interacting protein A; lpg2718), an effector protein originally identified because of its dependence on the IcmSW complex for translocation, but for which the function is unknown (14). The crystal structure reveals a two-domain structure comprising a metallophosphoesterase attached to an 85-Å-long α-helical hairpin forming a coiled-coil at its tip. Although WipA exhibits a serine/threonine phosphatase fold, it functions preferentially as a protein-tyrosine phosphatase (PTP), even though there are no structural similarities to the general PTP fold (15). Site-directed mutagenesis of key residues in the active site confirm the catalytic mechanism based on a binuclear catalytic center. The protein forms homodimers in solution, and the dimer is mediated by the tip of the helical hairpin domain. We anticipate that the helical hairpin forms an extended binding area for protein-protein interactions in the host cell.

## Results

### Purification of WipA and WipA fragments

**Figure 1.**
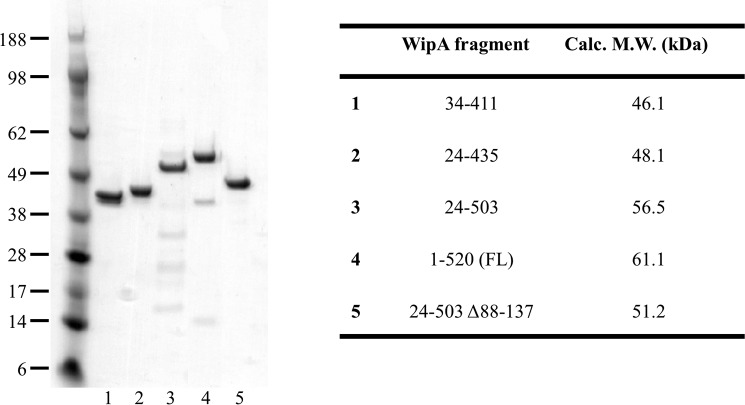
**Purified WipA proteins.** *Left panel*, SDS-PAGE of purified WipA fragments. *Right panel*, table with the corresponding molecular masses.

WipA (520 residues in total) was initially expressed as a fusion protein containing a decahistidine tag at the N terminus. The protein, however, degraded into shorter fragments, the most prominent of which had a molecular mass of 46 kDa. Mass spectrometry analysis of this fragment yielded a sequence coverage that included amino acids at the N and C termini from residue 34 to residue 411. A construct of this core domain (termed “WipA411”) was therefore produced, expressed, purified (Fig. 1), and crystallized, yielding crystals diffracting to 1.84 Å resolution. Its structure was determined using the single-wavelength anomalous dispersion method applied to selenomethionine (SeMet)-substituted crystals of WipA411 (Table 1). Residues including nine residues of the N-terminal His tag linker and the protein sequence up to residue 410 were visible in the electron density and were therefore included in the final model. The WipA411 crystals contain one molecule/asymmetric unit (AU) and no metal. Subsequently, a larger fragment, from residue 24 to residue 435 (termed “WipA435”) was produced containing a more complete active site. WipA435 crystallized in the presence of MnCl_2_ and crystals diffracted to a resolution of 1.75 Å. They contained two molecules/AU. The WipA435 structure was determined by molecular replacement using the WipA411 structure as a search model (Table 1). The model of WipA435 contains residues 24–418 in one molecule and residues 24–422 in the other. The two protomers of WipA435 superimpose with each other with an RMSD value of 0.72 Å, suggesting identical structures.

**Table 1:** **Data collection and refinement statistics** Information for the highest resolution shell is given in parentheses.

	WipA 33-411		WipA 24–435 (native)
	Native	SeMet	
**Data collection**			
Beamline	I02 (Diamond)	P13 (EMBL/PetraIII)	P13 (EMBL/PetraIII)
Wavelength (Å)	0.97949	0.9796	0.9999
Resolution range (Å)	80.69–1.84 (1.94–1.84)	81.17–2.40 (2.54–2.40)	46.5–1.75 (1.84–1.75)
Space group	C2	C2	P4_1_2_1_2
Cell parameters *a*, *b*, *c*, β (Å, grad)	103.06, 57.48, 80.71, 91.15	103.16, 58.27, 81.20, 91.73	80.65, 80.65, 321.98
Total reflections	127,897	115,421	785,524
Unique reflections	38,861	34,054	108,230
Multiplicity	3.3 (3.2)	3.4 (2.8)	7.3 (7.3)
Completeness (%)	94.5 (80.5)	91.9 (62.3)	99.9 (100.0)
Mean *I*/σ(*I*)	16.2 (1.58)	12.61 (0.96)	13.94 (1.25)
Wilson B-factor (Å^2^)	46.8	79.8	34.6
*R*_merge_ (%)	3.9 (95.1)	4.7 (73.8)	7.2 (135.6)
CC1/2	0.999 (0.900)	0.998 (0.603)	0.998 (0.555)

**Refinement**			
*R*_work_/*R*_free_ (%)	21.89/25.71		17.98/21.50
CC_work_/CC_free_	0.968/0.946		0.950/0.955
Protein atoms	3220		6631
Solvent molecules	154		629
Other atoms	4×acetate		4×Mn^2+^
			2×PO_4_^3+^
			6×Br^−^
			2×MPD
			1×βΜΕ
B-factor (Å^2^)			
Protein	60.91		43.20
Solvent	56.39		46.28
Ramachandran plot			
Favored (%)	96.88		97.77
Allowed (%)	3.12		2.23
Outliers (%)	0.00		0.0
Clash score	7.00		3.66
RMSD			
Bonds (Å)	0.005		0.007
Angles (grad)	1.323		1.085
PDB code	5N72		5N6X

Larger fragments of WipA have the propensity to degrade from the C terminus. Therefore, further attempts at producing these and the full-length protein focused on C-terminal uncleavable hexahistidine-tagged versions (supplemental Table S1). We were able to purify a larger fragment of WipA, from residue 24 to residue 503 (WipA503), as well as a more stable full-length protein (Fig. 1); however, none of them crystallized. These proteins and mutants thereof were, however, used for biochemical and activity assays.

### Overall structure

**Figure 2.**
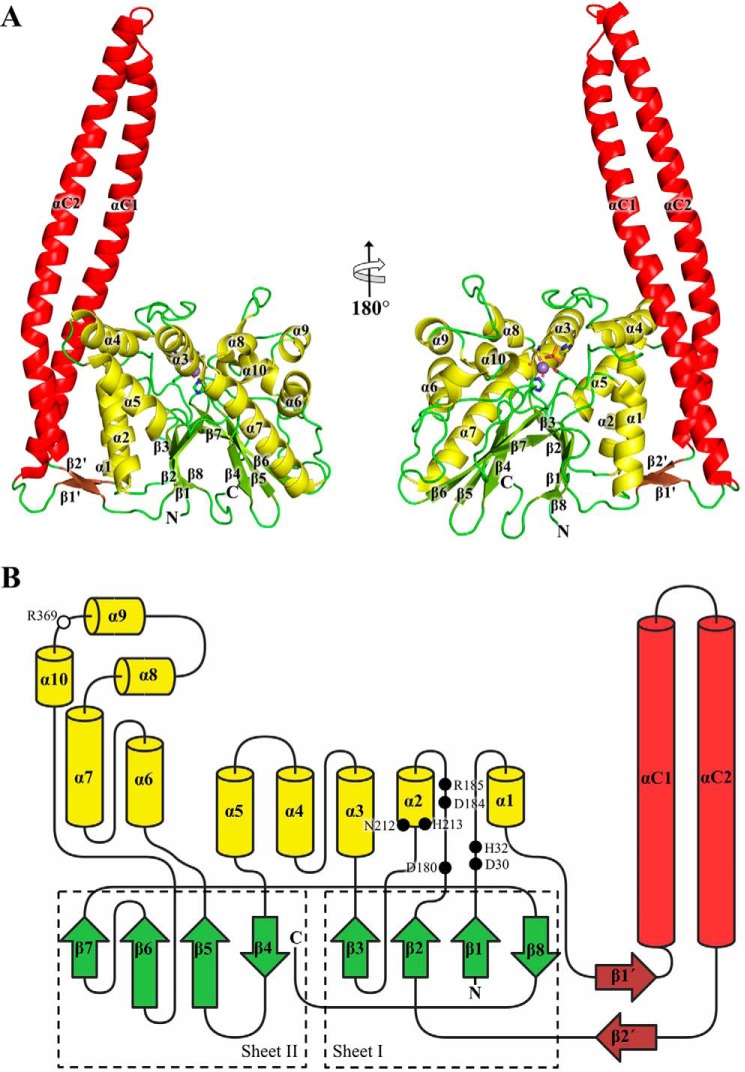
**The WipA structure and folding topology.** *A*, cartoon representation of the WipA435 structure in two orientations rotated by 180°. Color coding is based on the secondary structure elements and domains: α-helical hairpin in *red*, β-sheet linking the hairpin to the phosphatase domain in *brown*, remaining β-strands in *green*, and α-helices in *yellow*. The N and C termini of the protein and the secondary structure elements are labeled. The manganese ion is represented as a *purple sphere*, and the two coordinated water molecules are *small red spheres*. Key residues highlighting the active center are represented as *sticks*. In the panel at *right*, the edge strands β7 and β8 are clearly visible: they contain residues 411–422, *i.e.* the region missing in WipA411 but present in WipA435. *B*, the WipA435 folding topology. Color codes are as in *A*. The two central β-sheets are highlighted by *dashed boxes*. The positions of the phosphoesterase motifs residues are labeled as *solid black circles*. The Arg^369^ involved in the phosphotyrosine specificity is indicated with a *white circle*.

The description of the WipA structure focuses here on WipA435, the most complete structure obtained. The WipA monomer has overall dimensions of 104 × 66 × 44 Å and is comprised of two domains, a globular domain (in *green* and *yellow* in Fig. 2) flanked by an 85 Å long α-helical hairpin (in *red* in Fig. 2). The globular domain consists of a central β-sandwich flanked by α-helices (in *green* and *yellow*, respectively, in Fig. 2). Each β-sheet in the β-sandwich is formed of four β-strands, three of which are in a parallel configuration (β1, β2, and β3 in one β-sheet and β5, β6, and β7 in the other), whereas the fourth (β8 for the first sheet and β4 for the second) forms an antiparallel side strand (Fig. 2*B*). The β-sandwich is decorated by ten helices, five on either side of the central β-sandwich (in *yellow* in Fig. 2). Thus, except for strand β8, which is formed by the C-terminal end of the protein sequence, each β-sheet and their associated five helices are clearly partitioned along the protein sequence, with β1–3 and α1–5 formed by the N-terminal half of the protein, whereas the C-terminal half of the protein forms β4–7 and α6–10 (Fig. 2*B*).

**Figure 3.**
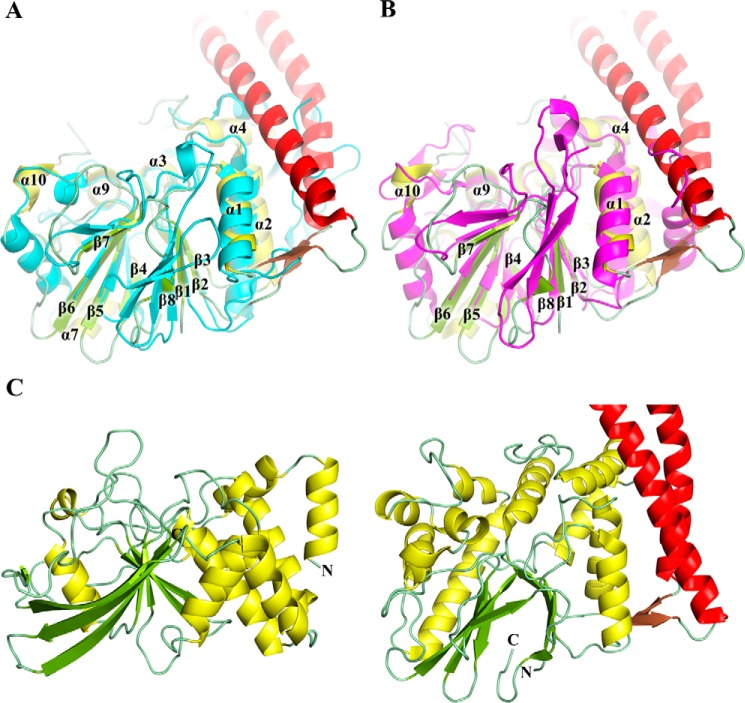
**Structural comparison of WipA with other phosphatases.** *A*, cartoon representation of superposed structures from WipA in same color coding as in Fig. 2 and the CAPTPase from *S. spongiae* in *cyan* (PDB code 1v73). Secondary structure elements of WipA common to both WipA and CAPTPase are labeled according to Fig. 2 (the conserved α5-helix is not shown in this orientation). *B*, cartoon representation of superposed WipA (color-coded and oriented as in *A*) and the human serine-threonine phosphatase PP1α in *magenta* (PDB code 3e7b). Secondary structure elements of WipA common to both WipA and PP1α are labeled (the conserved α5-helix is not shown in this orientation). *C*, comparison of an archetypical protein-tyrosine phosphatase, PTP1β (PDB code 1pty) (*left panel*) and WipA (*right panel*). Color coding of WipA is as in Fig. 2, whereas for PTP1β, color coding is *yellow* for helices and *green* for strands.

A search for structures homologous to the WipA globular domain in the Protein Data Bank using the DALI server (16) revealed more than 70 deposited entries with RMSD values between 2.0 and 3.0 Å. All domains belonged to the metallophosphoesterase family of proteins, which is a widely distributed superfamily among taxa (17), and require a variety of different metals for catalysis. The archetypal member of this family is the bacteriophage λ-phosphatase (17). This family of proteins includes notably a very large number of serine/threonine phosphatases including the PP1, PP2α, and PP5 subfamilies (Fig. 3, *A* and *B*, and supplemental Fig. S2). Among structural homologues, however, we identified one particularly intriguing and unique phosphatase, the cold-active PTP from *Shewanella spongiae* (PDB code 1v73; Fig. 3*B*) (18). The latter was the only PTP in the list of structural homologues returned by the DALI server. To date, *S. spongiae* cold-active PTP is the only member of the metallophosphoesterase family to have been shown to have tyrosine dephosphorylation activity. Classical PTP folds superpose poorly with either WipA435 or *S. spongiae* structures (Fig. 3*C*).

**Figure 4.**
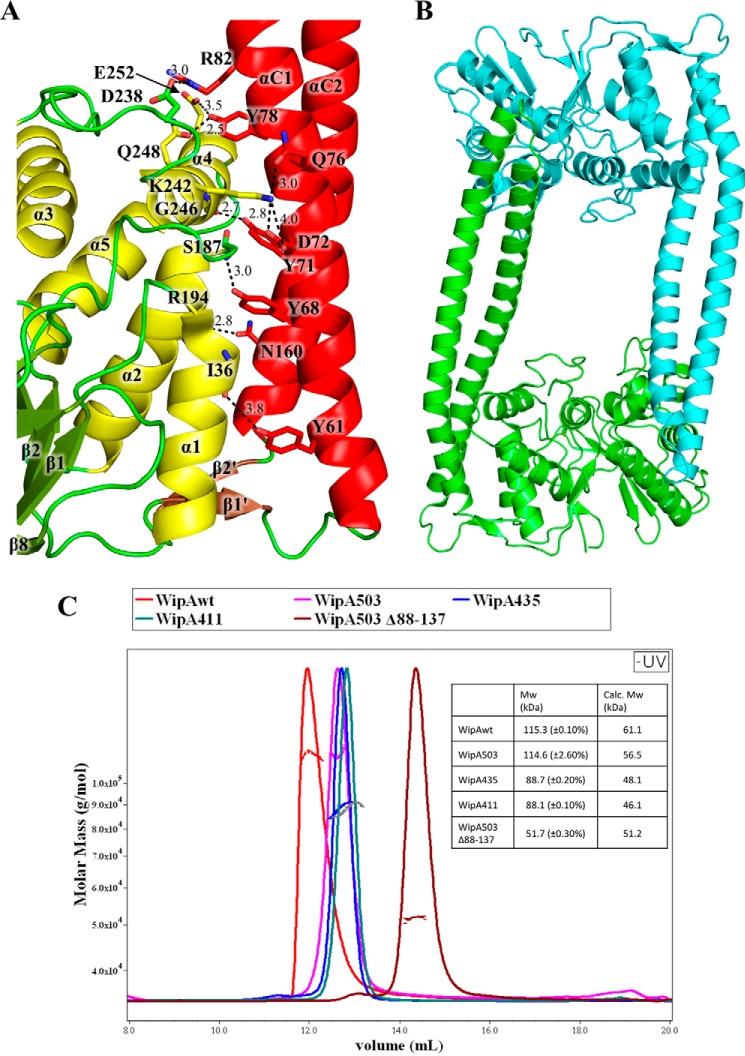
**WipA domain interfaces.** *A*, cartoon representation of the interface between the phosphatase domain and the α-helical hairpin in WipA. Key secondary structure elements of WipA are labeled, and color coding is as in Fig. 2. The interaction is based on an H-bond and salt-bridge network that is highlighted by *dashed lines. B*, cartoon representation of the functionally relevant dimer of WipA. This dimer is found in both WipA411 and WipA435 crystals. It is mediated by interaction between the coiled-coil region of the α-helical hairpin and the globular phosphatase domain, which was shown to be the relevant dimer in solution (see main text and *C*). *C*, SEC-MALS analysis of various WipA fragments. The *inset* reports on the MW measured experimentally using SEC-MALS and the molecular mass calculated from the sequence.

What makes the structure of WipA unique is the presence of the α-helical hairpin. This substructure is formed by a sequence inserted between the two strands of a small β-sheet (β1′ and β2′ colored in *brown* in Fig. 2) that links it to the globular domain. The WipA α-helical hairpin displays a 161° antiparallel left-handed twist spanning residues Asn^56^–Ala^109^ (helix αC1) and Gln^113^–Gln^165^ (helix αC2). Approximately one-third of the hairpin is interacting with the globular domain. The free-standing region of the α-helical hairpin (*i.e.* the region of the hairpin not interfacing with the globular domain) includes residues Leu^85^–Leu^106^ in the αC1 helix and Leu^120^–Leu^141^ in the αC2 helix, and this region of the hairpin forms a canonical coiled coil. The hairpin/globular domain interface includes eight Hydrogen bonds (H-bonds) and three salt bridges contributed by residues in α-helices α1, α2, and α4 (Fig. 4*A*). The 1,450 Å^2^ of the interface area represent ∼17% of the total hairpin surface.

### WipA forms dimers in solution

Both WipA crystal structures suggest interaction interfaces of potentially biological relevance (Fig. 4*B* and supplemental Fig. S1). The first is between the tip of the α-helical hairpin of one molecule and the globular domain from a symmetry-related molecule and is observed in both the WipA411 and WipA435 structures (supplemental Fig. S1, *A*, *left panel*, and interaction details in *B*). This interface includes five H-bonds and one salt bridge covering a surface area of ∼550 Å^2^. The second interface is observed only in the WipA435 crystals and involves a disulfide bridge from Cys^412^ and a Mn^2+^ ion coordinating two Asp^409^ residues from symmetrically related molecules (supplemental Fig. S1*A*, *right panel*). This interface, 780 Å^2^ in size, includes seven H-bonds. To identify whether any of these dimers are present also in solution, we ran a series of size-exclusion chromatography and multiangle light scattering (SEC-MALS) experiments using the full-length protein and different fragments thereof. Overall, all proteins including the shorter WipA411 elute as dimers (Fig. 4*C*). Thus, WipA is dimeric, and the interface mediating dimer formation is likely the region involving the coiled-coil tip of the α-helical domain. To test this hypothesis, we deleted this region including residues 88–137 in the WipA503 construct and conducted SEC-MALS experiments to test dimer formation. We observed that this fragment missing the part of the interacting region of the helical hairpin elutes as a monomer (Fig. 4*C*). These results suggest that the protein in solution forms an antiparallel dimer with the helical hairpin bridging two globular domains.

### The active site

**Figure 5.**
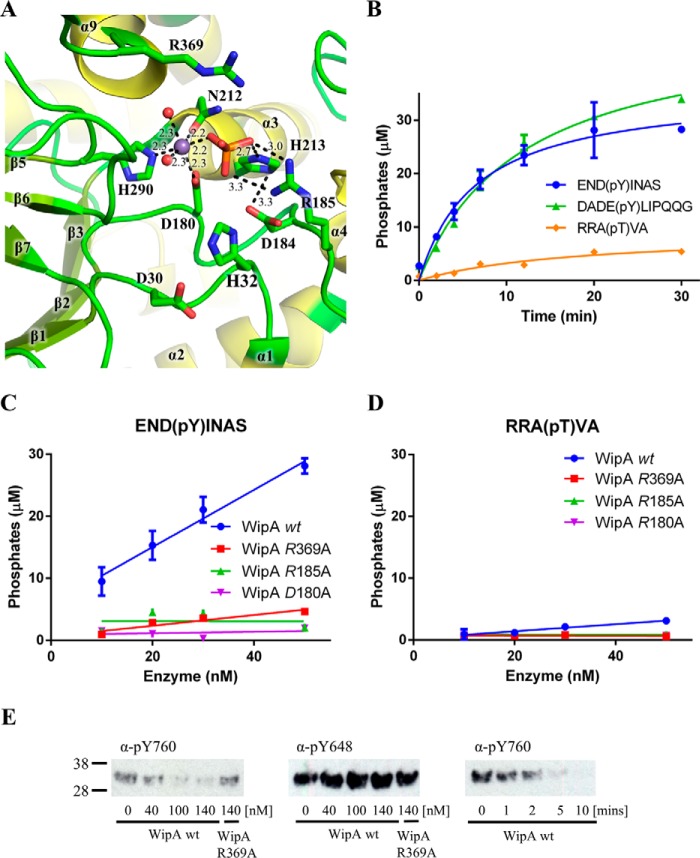
**WipA active site and phosphopeptide specificity.** *A*, cartoon representation of the active site following the color-coding of Fig. 2. The amino acids of the phosphoesterase motif are labeled, and H-bond interactions are represented by *dashed lines*. Distances for those bonds are reported. *B*, dephosphorylation of phosphotyrosine and phosphothreonine-containing peptides by WipA. Time courses of hydrolysis are shown using 20 nm full-length wild-type WipA. *C*, dephosphorylation of the phosphotyrosine peptide END(pY)INAS by WipA and WipA mutants. Phosphate release is plotted against increasing concentrations of WipA proteins. *D*, dephosphorylation of the phosphothreonine peptide RRA(pT)VA by WipA and WipA mutants. Phosphate release is plotted against increasing concentrations of WipA proteins. *E*, dephosphorylation of tyrosine-phosphorylated FGFR3 by WipA. The extent of tyrosine-phosphorylation of residue Tyr^760^ and Tyr^648^ in the FGFR3 kinase domain was monitored by Western blotting analysis followed by specific detection of the phosphorylation state of these residues using specific anti-Tyr(P)^760^ (α-*pY760*) and anti-Tyr(P)^648^ (α-*pY648*) antibodies (indicated for each panel). *Left panel*, from *left* to *right* the blots indicate the phosphorylation levels of the FGFR3 Tyr^760^ incubated at WipA concentrations of 0, 40, 100, and 140 nm; a control 140 nm WipA Arg^369^ is shown. *Middle panel*, same as in *left panel* but for phosphorylation levels of the FGFR3 Tyr^648^. *Right panel*, time course of phosphorylation levels of the FGRF3 Tyr^760^ incubated with 60 nm WipA at 0, 1, 2, 5, and 10 min.

The strong structural homology of WipA with metallophosphoesterase family proteins suggested that metal ions, including Mn^2+^, may be identified bound to WipA435. We therefore collected a data set at the Mn^2+^ K-edge (supplemental Table S2). A difference anomalous Fourier map revealed the presence of four Mn^2+^ ions: one in each molecule refined to full occupancy that appear strongly bound (Fig. 5*A*), two (one at the interface between two WipA molecules (supplemental Fig. S1*A*, *right panel*), and one in chain B in the proximity of the active site) appearing to be weakly bound. The strongly bound ions locate within proximity of three sequence motifs (D*X*H, GD*XX*DR, and GNHE) known to be signature motifs for metallophosphoesterases (17) (see below and supplemental Fig. S2), suggesting that these ions (one in each protomer) might be catalytic ions and that the residues around them might form the active site. A strong additional density was also observed near these Mn^2+^ ions; this density was present in the composite omit map (supplemental Fig. S1*C*) but not present in the anomalous Fourier map obtained at the Mn^2+^ edge. It was, however, larger in diameter than water and was interpreted as phosphate, which is frequently observed at this position in similar structures such as PP1α, PP2β, PP5, and λ-phosphatase (17, 19–21).

The D*X*H, GD*XX*DR, and GNHE sequences are located in the β1-α1 loop (Asp^30^ and His^32^), β2-α2 loop (Asp^180^, Asp^184^, and Arg^185^), and the α2 helix (Asn^212^ and His^213^), respectively (Fig. 2*B* and supplemental Fig. S2). All three sequences in the structure locate in one region of the structure, suggesting that the active site of WipA is located there (Fig. 5*A*). This region indeed comprises the one fully occupied Mn^2+^ cation described above in a tetrahedral bipyramidal arrangement coordinated by the WipA residues Asp^180^, His^290^ (a residue outside the phosphatase motifs), and Asn^212^, two water molecules, and a phosphate ion (Fig. 5*A*). The phosphate ion is observed interacting with His^213^, the imidazole group of which is acting as a proton carrier, supported in this role by the charge-relay residue Asp^184^. Two oxygens from the phosphate ion are further coordinated with the α-amino group of Arg^185^ (Fig. 5*A*). Finally, Arg^369^, a residue only conserved between WipA and the cold-active PTP from *Shewanella*, projects toward the phosphate ion but makes only a distant interaction with it (supplemental Fig. S3*A*).

To assess whether WipA has phosphatase activity, purified WipA and six mutants of the full-length protein were assayed for hydrolysis of 6,8-difluoro-4-methylumbelliferyl phosphate (DiFMUP) substrate in the presence of 2 mm Mn^2+^ using the EnzChek phosphatase assay kit from Molecular Probes, a kit particularly well suited to the monitoring of any phosphatase activities in any proteins (Table 2). Five of the six mutants were single-site mutants and included the mutations H32A, D180A, R185A, H213A, and R369A (see location of these residues in Fig. 5*A*). One additional mutant consisted of full-length WipA in which the 88–137 region of the helical hairpin was deleted: as shown above, this mutant is monomeric and was assayed for phosphatase activity to test whether dimerization affects WipA enzymatic activity.

**Table 2:** **Kinetic parameters for hydrolysis of DiMFUP** WipA was tested in the presence of Mn^2+^, Ni^2+^, Ca^2+^, and Mg^2+^. All WipA mutants were measured in the presence of Mn^2+^.

Protein	*K_m_*	*k*_cat_	*k*_cat_/*K_m_*
	*mm*	*min*^−*1*^	*min*^−*1*^·*mm*^−*1*^
WipA Δ88−137	0.0303 ± 0.0043	9.2802 ± 0.4275	306
WipA (Mn^2+^)	0.0113 ± 0.0008	2.435 ± 0.067	214.9
WipA (Ni^2+^)	0.0244 ± 0.0061	0.138 ± 0.011	5.66
WipA (Ca^2+^)			Inactive
WipA (Mg^2+^)			Inactive
WipA D180A			Inactive
WipA H32A			Inactive
WipA H213A	305.32 ± 19.64	151.6 ± 7.2	0.5
WipA R185A	0.0862 ± 0.0009	8.045 ± 0.335	93.4
WipA R369A	0.0461 ± 0.0029	3.218 ± 0.131	69.8

Because mutations may affect the stability of the mutated proteins and therefore affect the interpretation of activity assays, we first assessed the stability of the WipA mutants. We measured the melting temperatures (*T*_m_) in the presence or absence of Mn^2+^. Overall, the full-length protein in the SEC buffer shows a *T*_m_ in the range of 58–59 °C for most of the mutants except the WipA H213A that is slightly lower (56.62 °C). The addition of Mn^2+^ in the buffer resulted an increase of the *T*_m_ of ∼4° except from the WipA D180A and WipA H32A where the melting temperature was increased by 1–2°. Each of these two mutants would coordinate a metal ion, suggesting that the stabilizing effect of the Mn^2+^ is due to coordination in two different binding sites (Table 3).

**Table 3:** **Melting temperatures (*T*_m_) for different WipA proteins in presence/absence of MnCl_2_**

Protein	SEC buffer	SEC buffer + Mn^2+^
	°*C*	°*C*
WipA	58.49 ± 0.20	62.95 ± 0.16
WipA D180A	57.92 ± 0.11	58.81 ± 0.23
WipA H32A	58.31 ± 0.30	60.25 ± 0.23
WipA H213A	56.62 ± 0.06	62.36 ± 0.03
WipA R185A	58.42 ± 0.03	65.01 ± 0.17
WipA R369A	59.19 ± 0.30	63.02 ± 0.34

Knowing that the chosen mutations do not affect the structural integrity of WipA and WipA variants, we proceeded to carry out the activity assays. Any mutational effect that reduces the activity (*k*_cat_/*K_m_*) by >15% is defined as significant (22). Mutation D180A that is structurally important for Mn^2+^ binding displayed a complete loss of activity. Activity in H32A is also completely abrogated. H32A is part of the DH*X* motif, a motif previously characterized in phosphatases as being involved in binding of a second Mn^2+^ ion (supplemental Fig. S3*A*). In the *Shewanella* PTP structure, a Ca^2+^ ion used for crystallization was also observed bound to a similarly positioned His residue (supplemental Fig. S3*A*). Thus, His^32^ is an essential catalytic residue of WipA, likely because of its involvement in Mn^2+^ binding. The fact that we do not observe Mn^2+^ bound to His^32^ might be due to an incomplete structure lacking a large part of the C terminus of the full-length protein. This is consistent with WipA435 being inactive in the phosphatase assay (data not shown). The H213A mutation in WipA also yields a protein variant with significantly decreased activity, emphasizing the central role for this residue in catalysis. The R185A and R369A mutants displayed a loss of activity of ∼60–70%, suggesting non-essential yet important role in the reaction mechanism. Truncation of residues 88–137 in the α-helical hairpin has no impact on the activity of WipA, indicating that dimer formation does not play a role in WipA activity (Table 2).

The vast majority of structures returned by DALI as close homologues of WipA are serine/threonine phosphatases. Only one of the returned structures, that of the cold-active PTP from *Shewanella*, has tyrosine phosphatase activity, yet its structure is vastly different from classical PTP folds. To assess the specificity of WipA against phosphopeptides, phosphate release using the phosphotyrosine peptides END(pY)INAS and DADE(pY)LIPQQG, and the phosphothreonine peptide RRA(pT)VA as substrates was measured. Progress curves using a concentration of 20 nm WipA are shown in Fig. 5*B*. The results clearly demonstrate a much stronger activity toward tyrosine phosphopeptides compared with threonine phosphopeptides. Further analysis using various concentrations of WipA and plotting released phosphate concentration *versus* WipA concentration (Fig. 5, *C* and *D*, and supplemental Fig. S3*B*) confirm that WipA has phosphatase activity directed toward tyrosine-phosphorylated peptides. The activity was also assayed using the phosphatase-dead mutant D180A, and two additional mutants, R185A and R369A (Fig. 5, *C* and *D*, and supplemental Fig. S3*B*). In SH2 domains, domains known to specifically bind to tyrosine-phosphorylated peptides, two equivalent arginine residues make important interactions with the phosphotyrosine (23, 24): one (equivalent to Arg^185^ in WipA) makes direct bidentate interaction with the phosphate, whereas the other (equivalent to Arg^369^ in WipA) locates right above the phenyl ring of the phosphotyrosine and makes a distinct amino-aromatic interaction with it. Phosphotyrosine phosphatase activity is abolished in both the R185A and R369A WipA mutants (Fig. 5*C*), suggesting a potential similarity of interactions with phosphopeptides between WipA and SH2 domains. However, R185A and R369A WipA mutants are also inactive against a phosphothreonine peptide (Fig. 5*D*), although the activity of wild-type WipA against this peptide is low to start with, and thus, small reductions in activity might not be detectable. Finally, phosphotyrosine phosphatase activity was also tested in the mutant of WipA where residues 88–137 were deleted, and activity was shown to be comparable with that of wild-type (supplemental Fig. S3*C*), confirming that dimerization of WipA has no impact on WipA enzymatic activity. We note that the phosphatase activity of WipA (as measured using 6,8-difluoro-4-methylumbelliferyl phosphate (DiMFUP)) is ∼3 times lower than the well-characterized SHP2 under similar conditions and using the same assay (25). Thus, WipA activity is lower than in other PTPs but nevertheless is still significant.

We further tested the WipA phosphatase activity against the kinase domain of the fibroblast growth factor receptor 3 (FGFR3) protein. The FGFR3 kinase can be autophosphorylated at the amino acid positions Tyr^648^ and Tyr^760^ (26). Incubation of a FGFR3 protein phosphorylated at these positions with WipA clearly suggests a decrease of the phosphorylation levels at the position Tyr^760^ with both increasing concentrations and increasing incubation time of WipA wild-type (Fig. 5*E*, *left* and *right panels*). In contrast, no effect is observed for the Tyr(P)^648^ position (Fig. 5*E*, *middle panel*), indicating that WipA is selective for Tyr^760^ dephosphorylation.

## Discussion

WipA is a *Legionella* effector of unknown function, which was initially investigated for its IcmSW dependence for transport. We show here that WipA is an unusual protein-tyrosine phosphatase with a threonine/serine phosphatase fold mounted on a helical hairpin. The tip of the helical hairpin contains a canonical coiled-coil involved in the dimerization interface. Coiled coils are common sites of protein-protein interactions, mediating interaction with other functional domains including coiled-coils in protein partners. Interactions between coiled-coils are usually in the range of low nanomolar affinity (27) and obey general rules that are rather well characterized (28). The fact that the observed dimer interface is not mediated by interactions between the coiled-coil regions of two WipA molecules may thus indicate that the WipA dimer might be rather weak and that WipA might be able to transition between a dimeric form where the coiled-coil is weakly engaged with the globular phosphatase domain of another WipA molecule, as seen in the dimeric structure presented here, and a monomeric form where the coiled-coil is unmasked and available for engagement with other proteins, notably in the host. It has been noted previously that antiparallel coiled coils, rather than mediating homo-oligomerization, might be used more commonly to promote interactions with other proteins (29). We hypothesize here that the target of WipA in the host might well be a coiled-coil-containing protein whose activity is regulated by phosphorylation on tyrosine residues. Because the scope of the study presented here was to elucidate the structure and activity of WipA, we did not explore this hypothesis any further.

The catalytic domain of the WipA structure displays a clear resemblance to the phosphoesterase fold. WipA shows the highest activity when bound to Mn^2+^; however, it is still active in the presence of Ni^2+^, whereas no activity was observed when Ca^2+^ or Mg^2+^ were added in the buffer (Table 2). In general, enzymes of the phosphoesterase family can utilize a variety of different metals, even a combination of them (17). These enzymes are in general active against phosphoserine/threonine substrates, and indeed we have observed such an activity in WipA. Nevertheless, this activity was ∼7 times lower compared with WipA activity toward phosphotyrosine peptides. On the other hand, the vast majority of PTPs are based on the conserved cysteine sequence motif C*X*_5_R (15), and furthermore, most PTP structures contain a single central β-sheet, usually seven-stranded, irrespective of the catalytic residues involved in the catalysis (Fig. 3*C*). Although we initially hypothesized that a similar cysteine motif, around Cys^412^, might be responsible for the specificity of WipA toward phosphotyrosine, the structure clearly revealed that Cys^412^ is not located within the active site. Furthermore, the double central β-strand observed in WipA is very different from any known PTP fold. We conclude that the catalytic domain of WipA belongs to the metallophosphoesterase family yet supports an active PTP activity.

Such unusual PTP activity has been observed in the past for the cold-active PTP (CAPTPase) from *S. spongiae* (30). In this case, phosphotyrosine specificity was attributed to Tyr^161^; however, this residue is not conserved in WipA (the equivalently positioned residue in the structural-based alignment is Gln^240^) but is present at the same position in serine/threonine phosphatases (supplemental Fig. S2). Thus, this residue cannot be responsible for mediating specificity toward phosphotyrosine. In a search for possible residues that can contribute to specificity, we noticed that after the conserved motif LL(V)W in α-helix α9 there is an arginine at position 369 that is conserved in the CAPTPase but not in serine/threonine phosphatases (Fig. 3, *A* and *B*, and supplemental Fig. S2). Moreover, both WipA and CAPTPase contain bulky residues before and after the Arg^369^ that constrain and direct Arg^369^ toward the active center. Our observation that a WipA R369A mutant is inactive against phosphotyrosine-containing peptides suggests that Arg^369^ might confer PTP specificity. This role might be mediated by amino-aromatic interactions between the guanidinium group of the arginine and the aromatic group of the phosphotyrosine as observed previously in SH2 domains interaction with tyrosine-phosphorylated peptides (23, 24). However, this hypothesis should be moderated by the fact that R369A WipA appears to affect the phosphoserine/threonine activity of WipA, although this activity is very low to start with, and small activity differences might not be measurable at this very low level of wild-type activity. Thus, the enzymatic domain of WipA displays two activity determinants: the actual active site of the phosphoesterase domain that is hydrolyzing phosphates based on the well-known mechanism of the binuclear center and the surrounding active site residues that in the case of WipA suggest an unexpected substrate specificity toward phosphotyrosines. Remarkably, WipA is able to selectively dephosphorylate tyrosine residues within substrate proteins as demonstrated here for FGFR3. Indeed, we show here that only one of two phosphotyrosines within FGFR3 is dephosphorylated by WipA: it could be that the site protected from WipA activity might be less sterically accessible than the one targeted by WipA, or it could also be that WipA specifically targets sites with a defined sequence context around the phosphotyrosine residue.

Previous sequence analysis of WipA suggested the existence of two more paralogues, WipB and WipC, within *L. pneumophila*, and alignment of the sequences indicates that both contain the same phosphoesterase motifs (supplemental Fig. S4) (14). WipA differs from WipB and WipC in having the long sequence insertion corresponding to the α-helical hairpin, whereas WipB differs from WipA and WipC in having an additional C-terminal domain. WipC appears to be reduced to the sequence encoding the phosphatase activity. Although all three sequences are likely derived from a common ancestral gene, sequence insertions and deletions might confer various additional function to these proteins; because the enzymatic activity appears to be conserved in all three, it is tempting to propose that WipC is the ancestor and that the various sequences grafted onto WipC, *e.g.* the α-helical hairpin of WipA or the C-terminal domain of WipB, encode targeting functions that localize these proteins in different places with the host cell.

Future work on WipA will need to identify its target(s) in the eukaryotic host. We hypothesized above that this target could be coiled-coil-containing proteins subjected to regulation by tyrosine phosphorylation and dephosphorylation. WipA knock-out is unlikely to be phenotypically effective because most *Legionella* effectors appear to be highly redundant, and the presence of WipA paralogues certainly suggests a certain level of redundancy among WipA type of effectors. Nevertheless, one of the most exciting outcomes of research on *Legionella* pathogens is the unraveling of novel mechanisms to highjack and subvert a vast array of eukaryotic cell biological functions. In that regard, given the very distinct role tyrosine phosphorylation/dephosphorylation plays in higher eukaryotes, we would expect the discovery of novel targets of WipA to be just as exciting.

## Experimental procedures

### Cloning of WipA, WipA fragments, and WipA mutants

The WipA DNA (AAU28775) encoding the wild-type protein (Q5ZS02_LEGPH, lpg2718) was cloned in a modified pETM14 vector (EMBL) using a PCR-based in-fusion HD cloning system (Clontech). The expression cassette contained an N-terminal decahistidine tag followed by a 3C protease cleavage site. Additional constructs were generated from this original construct for different protein fragments, mutants, tags, and tag positions with the use of the in-fusion HD cloning system. Site-directed mutagenesis was performed using standard molecular biology protocols. Details for all generated constructs are given in supplemental Table S1.

### Expression and purification of WipA protein fragments

All recombinant proteins were overexpressed in C43(DE3) bacterial strains using a previously described autoinduction protocol (31). The cells were harvested by centrifugation (6000 × *g*, 15 min) and resuspended in a lysis buffer containing 25 mm Tris-HCl, pH 7.5, 0.3 m NaCl, 5 mm β-mercaptoethanol (βME), 10 mm imidazole, 5% glycerol, a tablet of protease inhibitors (Complete, EDTA-free by Roche), and 0.25 mg·ml^−1^ lysozyme. The cells were lysed in a EmulsiFlex-C3 homogenizer (Avestin), and the crude extract was centrifuged at 50,000 × *g* for 45 min. The supernatant was loaded onto a 5-ml HisTrap column (GE Healthcare) equilibrated with the lysis buffer, connected on an AKTA purifier (GE Healthcare). Washing steps were performed with extended volumes of lysis buffer though the column as well high-salt buffer (25 mm Tris-HCl, pH 7.5, 1 m NaCl, 5 mm βME, 10 mm imidazole, 5% glycerol). The protein was eluted using an imidazole gradient (elution buffer: 25 mm Tris-HCl, pH 7.5, 150 mm NaCl, 5 mm βME, 0.6 m imidazole, 5% glycerol). For the cleavage of the N-terminal decahistidine tag for WipA411 and WipA435, an on-column cleavage protocol was followed as described before (31). No His-tag removal was observed for the WipA411 fragment, suggesting that the 3C protease cleavage site is buried in the protein structure. The eluted protein was then dialyzed overnight against buffer A (25 mm Tris-HCl, pH 7.5, 5% glycerol) and loaded in a resource Q column (GE Healthcare). The protein was eluted with a gradient of NaCl at a concentration of ∼0.05–0.1 m NaCl. The eluted protein was further concentrated and loaded to a Superdex 200 16/60 column (GE Healthcare) equilibrated with a SEC buffer (25 mm Tris-HCl, pH 7.5, 0.15 m NaCl, 5 mm βME, 5% glycerol). Protein quality was assessed by SDS-PAGE. For most of the constructs the protein purity was over 95%, whereas an additional faint degradation band was always present for the wild-type protein.

To produce selenomethionine-containing WipA411 protein, the SelenoMet kit from Molecular Dimensions was used following a slightly modified protocol compared with the unlabeled protein. The expression plasmid was used to transform C43(DE3) cells, and a 50-ml overnight culture was used the next day to inoculate a 2-liter minimal medium culture containing vitamins and trace elements. After 2 h of the starvation step, all amino acids except methionine were added, and l-SeMet was added at a quantity of 40 mg·ml^−1^. The cells were induced with 0.5 mm isopropyl β-d-1-thiogalactopyranoside and further grown until the next morning reaching a final *A*_600_ over 2.0 at 20 °C. All following expression and purification steps were the same as for the non-labeled proteins.

### Protein stability assay

Protein stability of WipA and WipA mutants was assessed using a fluorescence thermal shift assay. The melting temperature (*T*_m_) for each protein was measured in a real-time PCR detection system IQ5 (Bio Rad) in a 96-well format according to literature (31). The volume for each reaction was 25 μl with a final protein concentration 4 μm and SYPRO Orange 8× (Molecular Probes) in the SEC buffer described above. The melting temperature defined as the inflection point of the melting curve was calculated by GraphPad Prism using a sigmoidal model.

### Protein crystallization

Initial crystallization screens were performed using the sitting-drop vapor-diffusion technique, by mixing equal volumes (0.2 μl) of protein solution (13 mg·ml^−1^ in SEC buffer containing in addition 2 mm MnCl_2_ for the case of WipA435) and reservoir solution from commercial crystallization screens at 16 °C. Crystals for both proteins (WipA411 and WipA435) appeared after 1–2 days reaching a maximum length of 0.3–0.6 mm in 5–7 days. The best crystals for WipA411 were observed in a precipitant containing 0.2 m sodium acetate and 18% PEG 3350. The best crystals for WipA435 were obtained using 0.1 m Bis-Tris propane, pH 7.5, 0.2 m NaBr, and 20% PEG 3350. Before data collection, harvested crystals were immersed in a solution containing the precipitant mixture and 10% 2-methyl-2,4-pentanediol (MDP) and cryo-cooled in liquid nitrogen.

### Data collection and refinement

All data sets were collected at 100 K. Crystals of the native WipA411 were measured at the I02 Beamline (Diamond), whereas all the remaining data sets were collected at the PetraIII P13 Beamline (EMBL-Hamburg/DESY) (32). All native data sets and the SeMet WipA411 data set were indexed, processed, and scaled using the XDS package (33) (Table 1). To assess the identity of bound metal ions in the WipA435 crystals, one more data set was collected at the Mn^2+^ K-edge (supplemental Table S2). This data set was processed by XDS and scaled by AIMLESS (34). The anomalous difference Fourier map was generated using the anomalous structure factors and calculated phases from the model using the programs SFALL and FFT in CCP4 (35).

The WipA411 crystals belonged to the *C* 2 space group with a solvent content of 54.5% corresponding to one molecule/AU. The WipA435 crystals belonged to the P4_1_2_1_2 space group with a solvent content of 57.2% corresponding to two molecules/AU. Single-wavelength anomalous dispersion phases from the WipA411 data set were derived using the SHELX suite (36) as implemented in the HKL2MAP interface (37). These phases were used to calculate an electron density map to a resolution of 2.4 Å. A preliminary model encompassing 80% of the structure was built using the ARP/wARP suite (38). This model was then used as a starting model for refinement using the high-resolution native data set of WipA411. After several iterations of rigid-body, maximum-likelihood, and TLS refinement using the PHENIX suite (39), manual building, and model inspection using COOT (40), the final model converged to a final *R*_work_/*R*_free_ of 0.2189/0.2571 at a resolution of 1.84 Å. The WipA411 model covers the WipA sequence from residues 33 up to 410, and a large part of the linker connecting the His tag to the protein sequence was visible in the electron density.

The WipA435 structure was determined by molecular replacement using MOLREP (41) and the WipA411 coordinates as reference model. The coordinates were further improved by maximum-likelihood and TLS refinement using the PHENIX suite and manual improvements of the model using COOT. The final model converged to a final *R*_work_/*R*_free_ of 0.1798/0.2150 at a resolution of 1.75 Å. The WipA435 model covers the WipA amino acid sequence 24–418 (chain A) and 24–422 (chain B) with the last 17 (chain A) and 13 (chain B) amino acids being disordered in the crystal. The model also contains 629 water molecules, four Mn^2+^ ions, two phosphate ions, six Br^−^ ions, two MPD molecules, and one βME molecule (Table 1).

### Data analysis and bioinformatics

Analysis of the protein-protein interfaces was performed by the online server PDBePISA(EMBL-EBI) (42). Structural alignment of the WipA structures with known PDB entries (Fig. 3) and sequence alignment and assignment of secondary structural elements (supplemental Fig. S2) were performed by PDBeFOLD(former ssm/EMBL-EBI) (43). The analysis of the coiled-coil regions was performed by the SOCKET server (44). Representation of sequence alignments was performed by the ESPript server (45). Molecular visualization of the crystal structures was done by PyMOL.

### Analytical SEC-MALS

SEC was performed using a Superdex 200 Increase 10/300 GL column (GE Healthcare) equilibrated in the SEC buffer described above. Separations were performed at 20 °C with a flow rate of 0.6 ml·min^−1^ using HPLC (Agilent Technologies 1100 series). The samples (100 μl) were injected at a concentration of 2 mg ml^−1^. Online MALS detection was performed with a dawn 8+ detector (Wyatt Technology Corp., Santa Barbara, CA) using a laser emitting at 690 nm and by refractive index measurement using an Optilab T-rex (Wyatt Technology Corp., Santa Barbara, CA). Data analyses were performed as previously described (46).

### Activity assays

To determine the Michaelis-Menten parameters we used the EnzCheck® (Molecular Probes) continuous spectrophotometric assay (47). Specific activity toward dephosphorylated DiMFUP was measured in a 96-well plate in 100 μl of total volume. The measurements were performed in the plate reader Synergy 2 (Biotek) using the Tungsten lamp and excitation/emission filters set at 360/460 nm. Each reaction was initiated after mixing equal volumes of DiMFUP at concentrations 5, 10, 30, 50, 80, and 100 μm in a 0.1 m acetate buffer, pH 5.0, and enzyme in a SEC buffer containing in addition 2 mm MnCl_2_. All reagents were pre-equilibrated at 37 °C, which was the temperature of the reaction. The release of the dephosphorylated DiMFUP was monitored every 10 s. To calculate the parameters V_max_ and *K_m_* in the Michalis-Menten equation, a non-linear regression model with unweighted uncertainty for all data was applied, using the Solver supplement of Microsoft Office Excel as previously described (48). The validity of the data were confirmed independently using the software GraphPad Prism.

To determine the specificity of WipA against phosphopeptides, the tyrosine phosphatase and serine/threonine phosphatase assays systems from Promega were used. All reagents were pre-equilibrated at 37 °C, the temperature at which the reactions were performed. The enzymatic reaction was initiated by adding 5 nmol of each phosphopeptide in a WipA solution in SEC buffer containing in addition 2 mm MnCl_2_. The reaction was stopped at specific time intervals by adding equal volume of the molybdate dye/additive mixture supplied by the Promega kits. The absorbance at 600 nm was measured in the same Synergy2 plate reader as above, and it was directly linked to the amount of the released phosphate ions using reference phosphate concentrations in the SEC/MnCl_2_ buffer. Blanks and control were measured in the absence of WipA and in the absence of buffers.

To determine whether WipA is active on tyrosine-phosphorylated proteins (as opposed to tyrosine-phosphorylated peptides as described above), the kinase domain of the FGFR3 covering the residues 455–768 was purified as described previously in an unphosphorylated state (26). For the protein phosphorylation, ∼60 μg of the kinase domain were incubated for 45 min in a buffer containing 25 mm Tris-HCl, pH 8.0, 150 mm NaCl, 10 mm MgCl_2_, 1 mm MnCl_2_, 2 mm NaVO_4_, 0.1 mm TCEP (kinase buffer), and 1 mm ATP in a total volume of 100 μl. To remove the remaining ATP, the reaction mixture was then passed through a PD-SpinTrap G-25 column (GE Healthcare) equilibrated with the reaction buffer (25 mm Tris-HCl, pH 8.0, 150 mm NaCl, 1 mm MnCl_2_, 0.1 mm TCEP). For the dephosphorylation assay FGFR3 kinase aliquots of ∼0.35 μg·μl^−1^ were incubated for 2 min with WipA at concentrations 0, 40, 100, and 140 nm at 37 °C. Α sample containing the WipA R369A at 140 nm was used as a negative control. The reaction was stopped by addition of SDS-PAGE loading buffer and incubation for 5 min at 70 °C. The samples were then analyzed on a 4–12% SDS-PAGE. The phosphorylated tyrosines in FGFR3 were identified through immunoblotting using antibodies recognizing FGFR1 phosphorylated tyrosines 654 (ab70959; AbCam) and 766 (ab59180; AbCam) that recognize FGFR3 phosphotyrosines 648 and 760, respectively. Bands were detected with ECL Prime (GE Healthcare, Amersham Biosciences) and imaged using Hyperfilm ECL (GE Healthcare, Amersham Biosciences). The figures are representative of three independent assays.

## Author contributions

N. P. conducted experiments, analyzed the results, and wrote the paper. G. W. wrote the paper with N. P. and supervised the experimental work.

## Supplementary Material

Supplemental Data

## Footnotes

2 The abbreviations used are:

T4bSS: type IVb secretion system
RMSD: root mean square deviation
PDB: Protein Data Bank
DiMFUP: 6,8-difluoro-4-methylumbelliferyl phosphate
PTP: protein-tyrosine phosphatase
CAPTPase: cold-active PTP
SeMet: selenomethionine
AU: asymmetric unit
SEC-MALS: size-exclusion chromatography and multiangle light scattering
FGFR: fibroblast growth factor receptor
βME: β-mercaptoethanol
SEC: size exclusion chromatography.
