# Supplementary material for: Structure of the WipA protein reveals a novel tyrosine protein phosphatase effector from Legionella pneumophila
Source: J Biol Chem. 2017 Apr 7;292(22):9240–51. doi: 10.1074/jbc.M117.781948 (PMC5454105; doi:10.1074/jbc.M117.781948)
Supplement: Supplemental Data [file supp_292_22_9240__index.html]

Structure of the WipA protein reveals a novel tyrosine protein phosphatase effector from Legionella pneumophila — Structure of the WipA protein reveals a novel tyrosine protein phosphatase effector from Legionella pneumophila — Crystal structure of the Legionella effector WipA — Supplemental Data 

# Structure of the WipA protein reveals a novel tyrosine protein phosphatase effector from *Legionella pneumophila*

## Supplemental Data

- Supplement data (.pdf, 4.7 MB) - With corrections based on the reviewers comments
