# Supplementary material for: Structure of the WipA protein reveals a novel tyrosine protein phosphatase effector from Legionella pneumophila
Source: J Biol Chem. 2017 Apr 7;292(22):9240–51. doi: 10.1074/jbc.M117.781948 (PMC5454105; doi:10.1074/jbc.M117.781948)
Supplement: Supplemental Data [file 10.1074_M117.781948_jbc.M117.781948-1.pdf]

Structure of the WipA protein reveals a novel tyrosine protein phosphatase effector from *Legionella pneumophila*

Nikos Pinotsis[1] and Gabriel Waksman[1,2*]

[1]Institute of Structural and Molecular Biology, Department of Biological Sciences, Birkbeck, Malet Street, WC1E 7HX London, United Kingdom
[2]Institute of Structural and Molecular Biology, University College London, Gower Street, WC1E 6BT London, United Kingdom.

Running title: Crystal structure of the *Legionella* effector WipA

* To whom correspondence should be addressed: Telephone: +44 020 7631 6833; E-mail: g.waksman@ucl.ac.uk and g.waksman@mail.cryst.bbk.ac.uk

Keywords: *Legionella* effector, tyrosine phosphatase, phosphoesterase fold, coiled-coil, crystal structure

Table S1. Proteins generated in this study

	**Name**	**Seq. range**	**Tag**	**MW (KDa)***
1	WipA411	35-411	N-term 10xHis	46.1
2	WipA435	24-435	N-term 10xHis	48.1
3	WipA503	24-503	C-term 6xHis	56.5
4	WipA503 Δ88-137	24-503, Δ88-137	C-term 6xHis	51.2
5	WipA*wt*	1-520	C-term 6xHis	61.1
6	WipA D180A	1-520	C-term 6xHis	61.1
7	WipA H213A	1-520	C-term 6xHis	61.1
8	WipA R185A	1-520	C-term 6xHis	61.1
9	WipA R365A	1-520	C-term 6xHis	61.1
10	WipA H32A	1-520	C-term 6xHis	61.1
11	WipA Δ88-137	1-520, Δ88-137	C-term 6xHis	55.8

*Including the tag sequence in all proteins except WipA435 that was cleaved

Table S2: Data collection statistics for the Mn^{2+} anomalous difference Fourier map for the WipA24-435 native crystals. Information for the highest resolution shell is given in parentheses

	**Mn^{2+} edge**
**Data Collection**	
Beamline	P13 (EMBL/PetraIII)
Wavelength (Å)	1.88425
Resolution Range (Å)	46.53 – 2.46 (2.56 – 2.46)
Space group	$P\ 4_1\ 2_1\ 2$
Cell parameters a=b, c (Å)	80.61, 322.21
Total reflections	984,827
Unique reflections	39,922
Multiplicity	24.7 (23.5)
Anomalous multiplicity	13.3 (13.2)
Completeness (%)	99.9 (99.6)
Anomalous completeness (%)	99.9 (99.4)
Mean I/Sigma(I)	31.3 (5.9)
Wilson B-factor (Å^2)	52.28
R_{merge} (%)	8.4 (58.9)
CC1/2	1.000 (0.955)

FIGURE S1. **Cartoon representation of the two major WipA interfaces in the crystal.** *A*, the dimeric WipA found in the asymmetric unit (AU) of the WipA435 crystal in green and cyan (left panel). In light gray is shown a WipA molecule from an adjacent AU that forms the second interface with the cyan WipA protomer. In the right panel the same assembly is rotated by 90 degrees highlighting the same cyan protomer as in the left panel and the WipA from the same adjacent AU as in the left panel but now colored in green. The Mn^{2+} ion is shown as a sphere colored in black. The box in black identifies the dimer interface shown in details in panel B. *B*, details of the interactions between WipA monomers. Of the two dimer interfaces identified in this study, only the one presented in this panel proved to be relevant in solution (see main text and Figure 4). The interface is based on a mixed H-bond and hydrophobic network. *C*, composite omit map at the WipA active site. The composite omit map is contoured at 1.0 σ and was generated using PHENIX with 5% of the atoms omitted per cycle. Protein residues and the phosphate ion are labeled. Water molecules are represented as red spheres and the Mn^{2+} ion as magenta sphere.

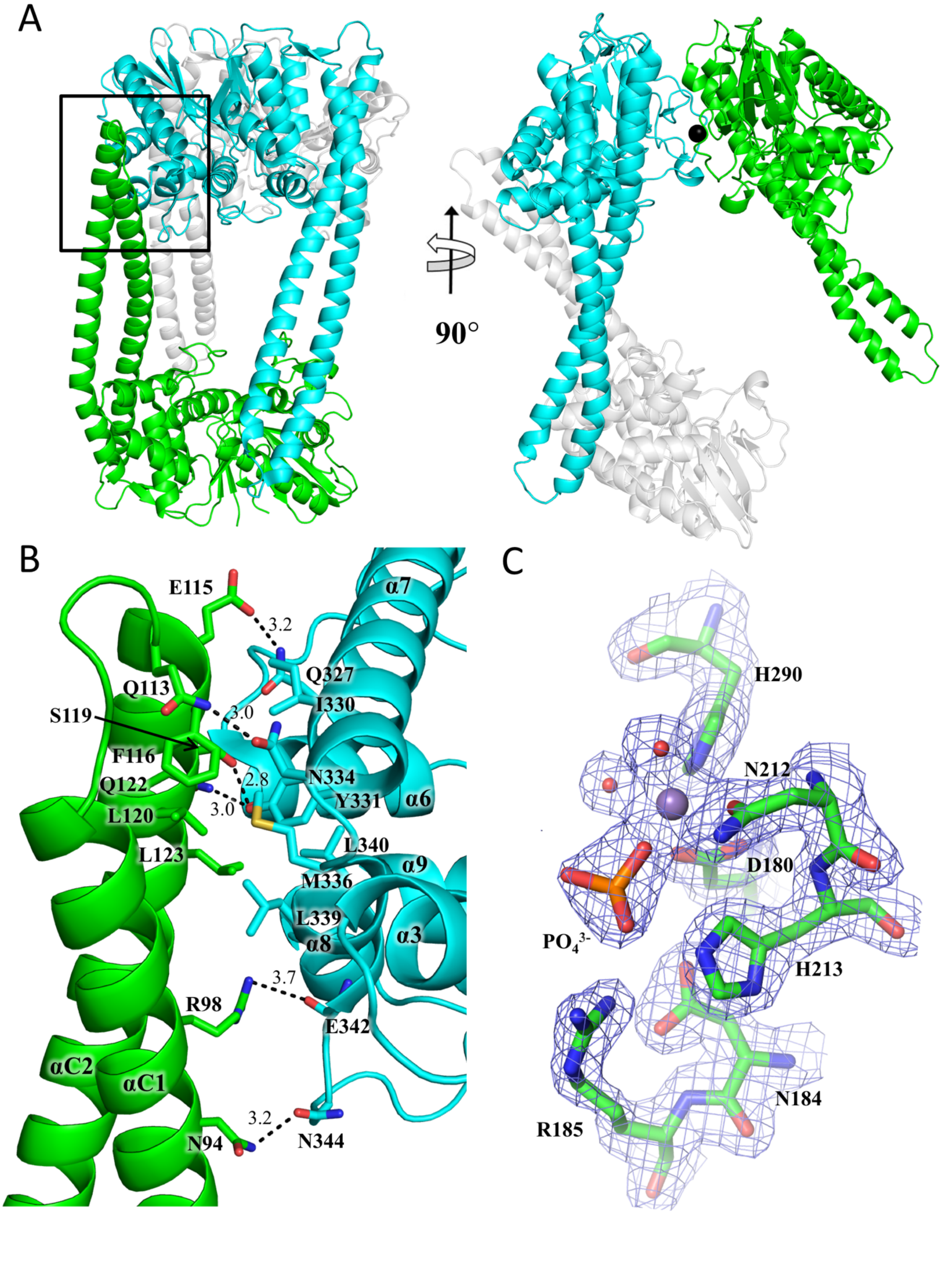
A
90°
B
E115
3.2
Q327
Q113
I330
S119
3.0
F116
2.8
N334
Q122
Y331
α7
α6
3.0
L120
L340
L123
α9
M336
L339
α8
α3
R98
3.7
E342
αC2
αC1
3.2
N94
N344
C
H290
N212
D180
PO_4^{3-}
H213
N184
R185

FIGURE S2. **Structure sequence alignment of WipA with similar phosphatases.** The aligned structures are as follows, WipA, CAPTPase the cold-active protein tyrosine phosphatase from *Shewanella spongiae* (PDB ID 1v73) (1), PP1 the human serine-threonine phosphatase PP1α (PDB ID 3e7b) (2), PP2 the human serine-threonine phosphatase PP2α (PDB ID 3fga) (3) and PP5 the human serine-threonine phosphatase PP5 (PDB ID 1s95) (4). Identical residues are boxed in red background and conserved residues are boxed and highlighted in red color. The secondary structure elements above the aligned sequences generated by DSSP (5) for the WipA435 structure. The three phosphoesterase motifs are highlighted underneath the aligned sequences in bold.

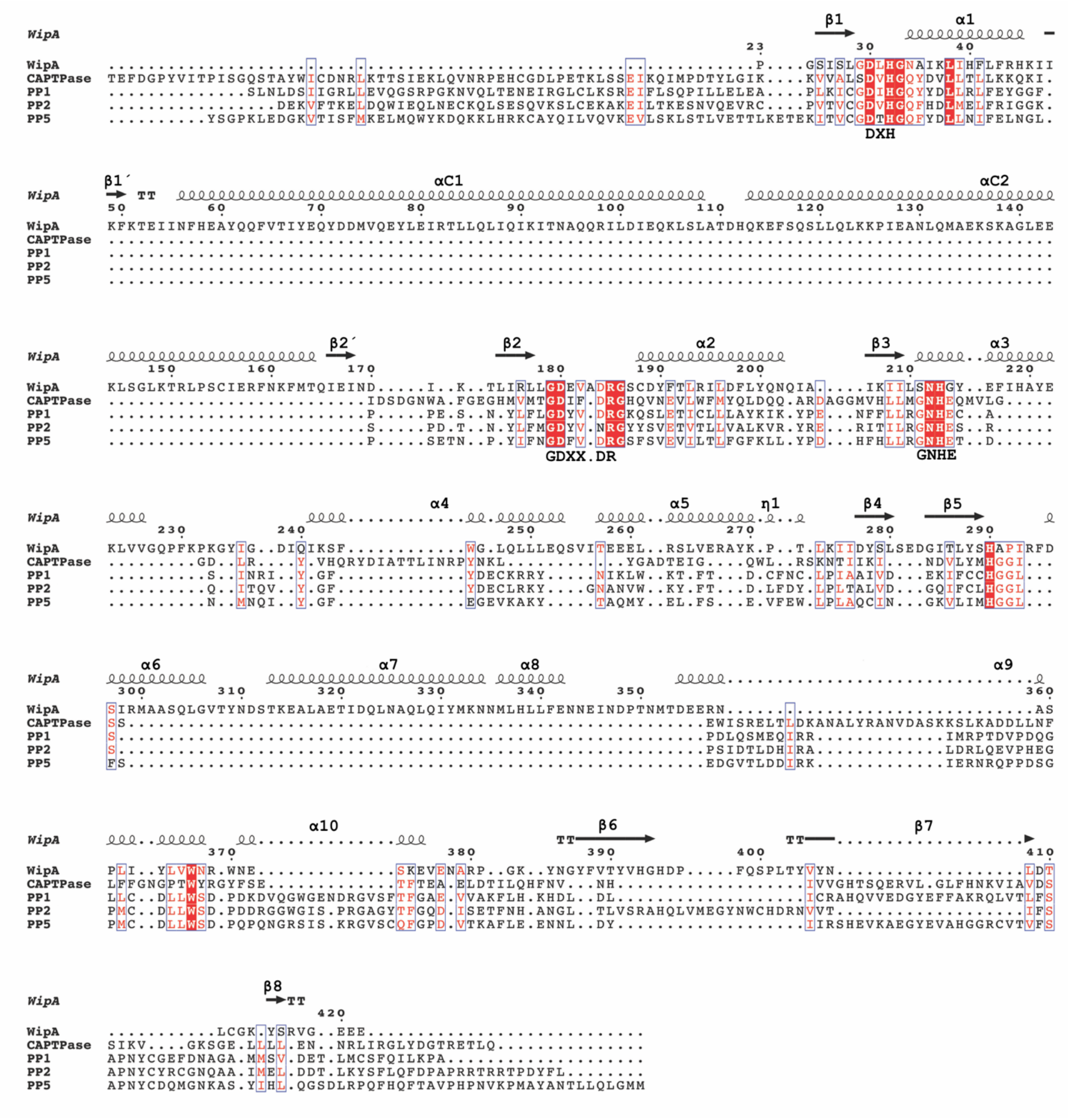

FIGURE S3. **Superimposition of the active sites from WipA, CAPTP and PP1α, and kinetics of dephosphorylation of tyrosine-phosphorylated peptides.** *A*, superimposition of the active sites from WipA, CAPTP and PP1α. Ribbon and stick representation of the active site of WipA in green, CAPTP in cyan (PDB ID 1v73) and PP1α in magenta (PDB ID 3e7b). The structures are aligned using three aminoacids of WipA, N212, H213 and D180. Mn^{2+} ion of WipA is shown as sphere superimposing with the Mn^{2+} from PP1α and the Ca^{2+} from CAPTP. The large conformational changes of WipA residues D30, H32 and H395 due to the missing second ion in the WipA active site are highlighted by black arrows. *B*, hydrolysis of phosphotyrosine-containing peptide DADE(pY)LIPQQG against increasing concentrations of WipA proteins. *C*, hydrolysis of phosphotyrosine-containing peptides against increasing WipA Δ88-137 concentrations.

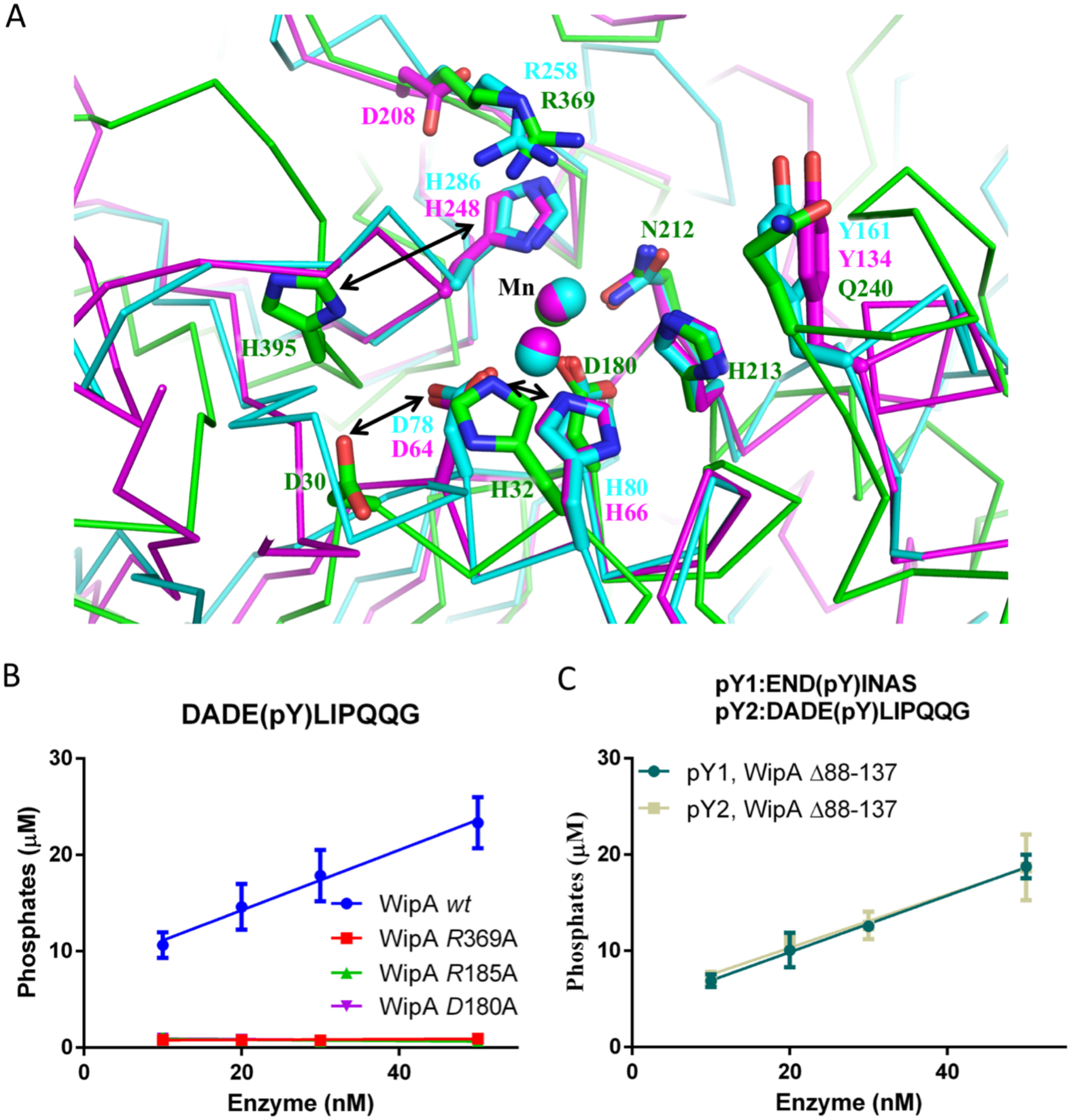

FIGURE S4. **Sequence alignment of *Legionella proteins* WipA(lpg2718), WipB(lpg0642) and WipC(lpg2206).** Identical residues are boxed in red background and conserved residues are boxed and highlighted in red color. The three phosphoesterase motifs are highlighted underneath the aligned sequences in bold.

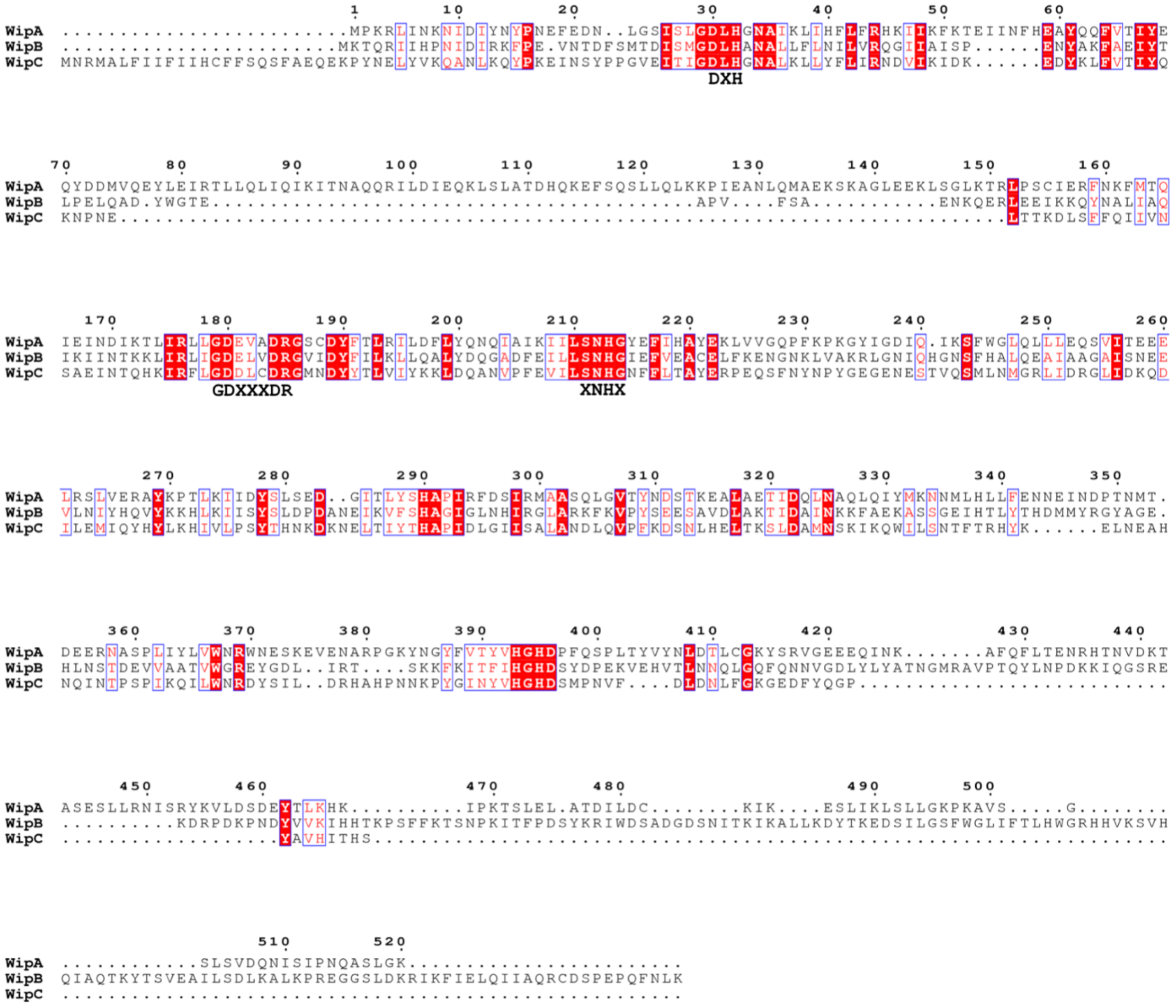

REFERENCES

1. Tsuruta, H., Mikami, B., and Aizono, Y. (2005) Crystal structure of cold-active protein-tyrosine phosphatase from a psychrophile, Shewanella sp. *Journal of biochemistry* **137**, 69-77
2. Kelker, M. S., Page, R., and Peti, W. (2009) Crystal structures of protein phosphatase-1 bound to nodularin-R and tautomycin: a novel scaffold for structure-based drug design of serine/threonine phosphatase inhibitors. *Journal of molecular biology* **385**, 11-21
3. Xu, Z., Cetin, B., Anger, M., Cho, U. S., Helmhart, W., Nasmyth, K., and Xu, W. (2009) Structure and function of the PP2A-shugoshin interaction. *Molecular cell* **35**, 426-441
4. Swingle, M. R., Honkanen, R. E., and Ciszak, E. M. (2004) Structural basis for the catalytic activity of human serine/threonine protein phosphatase-5. *J Biol Chem* **279**, 33992-33999
5. Kabsch, W., and Sander, C. (1983) Dictionary of protein secondary structure: pattern recognition of hydrogen-bonded and geometrical features. *Biopolymers* **22**, 2577-2637
